# Supplementary material for: A RAB7A phosphoswitch coordinates Rubicon Homology protein regulation of Parkin-dependent mitophagy
Source: J Cell Biol. 2024 May 10;223(7):e202309015. doi: 10.1083/jcb.202309015 (PMC11090050; doi:10.1083/jcb.202309015)
Supplement: Table S1 — shows the oligonucleotides for CRISPR KO cell lines. [file JCB_202309015_TableS1.docx]

**Table S1. Oligonucleotides for CRISPR KO cell lines**

| **Oligonucleotides** |  |
| --- | --- |
| RUBICON (genotyping) F | GCTGGGACTAAGGCATGTCC |
| RUBICON (genotyping) R | GTGGCCTGGACCAATGGG |
| RUBICON sequencing primer | ATTGTTTGTAGAGATGG |
| PACER (genotyping) F | CCAATTTGAGTTGTCCGTTG |
| PACER (genotyping) R | GAATATATGCCCCGTGGATAAG |
| PACER sequencing primer | GTAAGCCGAATCTATGTC |
