# Supplementary material for: A RAB7A phosphoswitch coordinates Rubicon Homology protein regulation of Parkin-dependent mitophagy
Source: J Cell Biol. 2024 May 10;223(7):e202309015. doi: 10.1083/jcb.202309015 (PMC11090050; doi:10.1083/jcb.202309015)
Supplement: Table S2 — shows the details of CRISPR sequences and genotyping results of knockout cell lines in this study. [file JCB_202309015_TableS2.docx]

**Table S2.** **Details of CRISPR sequences, genotyping results of knockout cell lines in this study**

| **Name** | **Gene symbol** | **Uniprot** | **GeneID/Location** | **Targeting strategy** | **CRISPR gRNA (PAM)** | **Clone** | **Unique alleles** | **Editing results** |
| --- | --- | --- | --- | --- | --- | --- | --- | --- |
| RUBICON KO | RUBCN | Q92622 | 9711/ NC_000003.12 | Exon 3 | ACTGCCAGTAATCCGTCTGG(CGG) | 15 | 1 | 1 bp deletion |
|  |  |  |  |  |  | 16 | 2 | 4 bp deletion, 1 bp insertion |
| PACER KO | RUBCNL | Q9H714 | 80183/ NC_000013.11 | Exon 5 | AGATATGATTATATCAGCAA(TGG) | 14 | 1 | 2 bp deletion |
|  |  |  |  |  |  | 17 | 1 | 1 bp deletion |
