# Supplementary figures and images for: A RAB7A phosphoswitch coordinates Rubicon Homology protein regulation of Parkin-dependent mitophagy
Source: J Cell Biol. 2024 May 10;223(7):e202309015. doi: 10.1083/jcb.202309015 (PMC11090050; doi:10.1083/jcb.202309015)

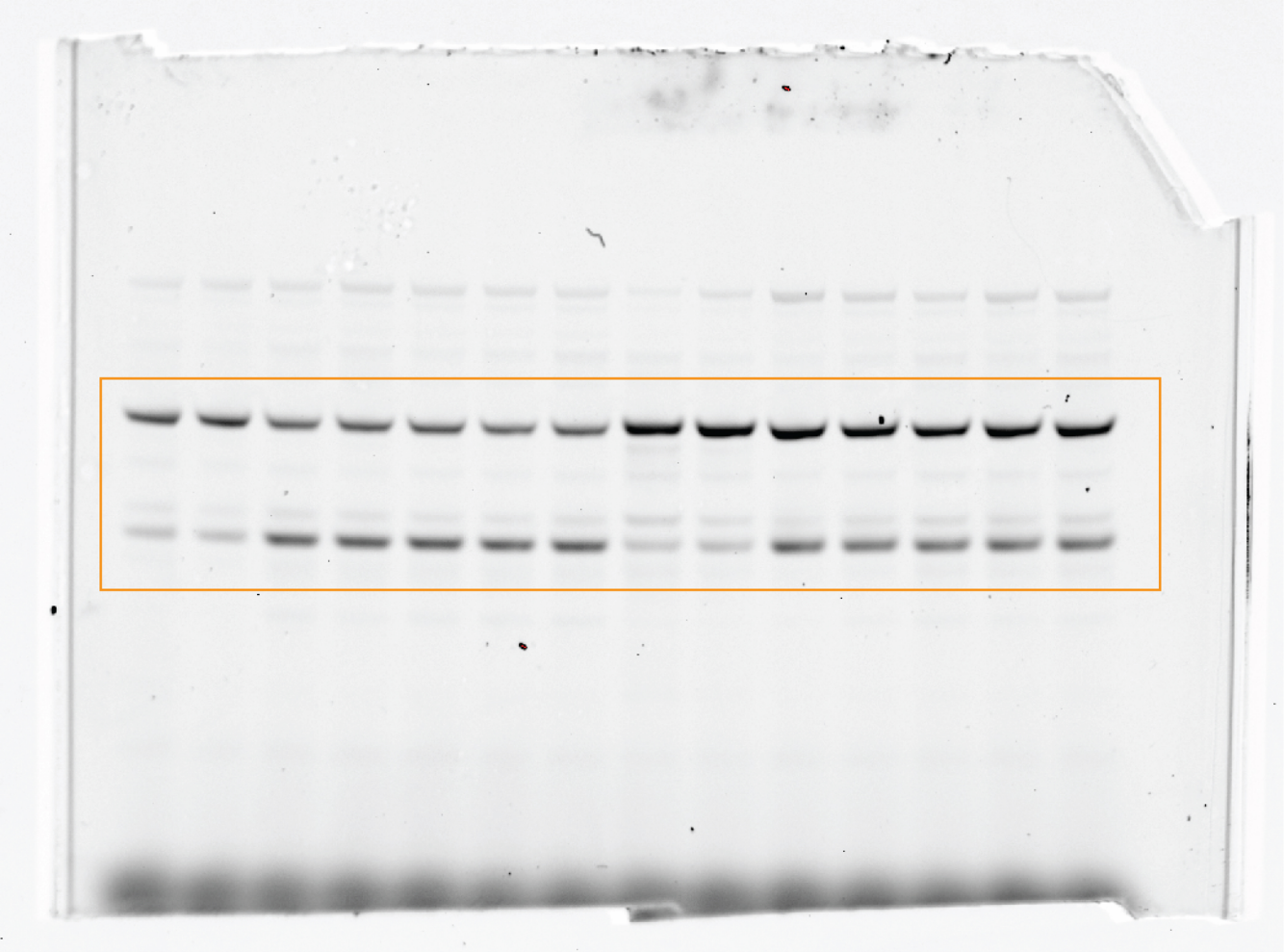

Figure 1A

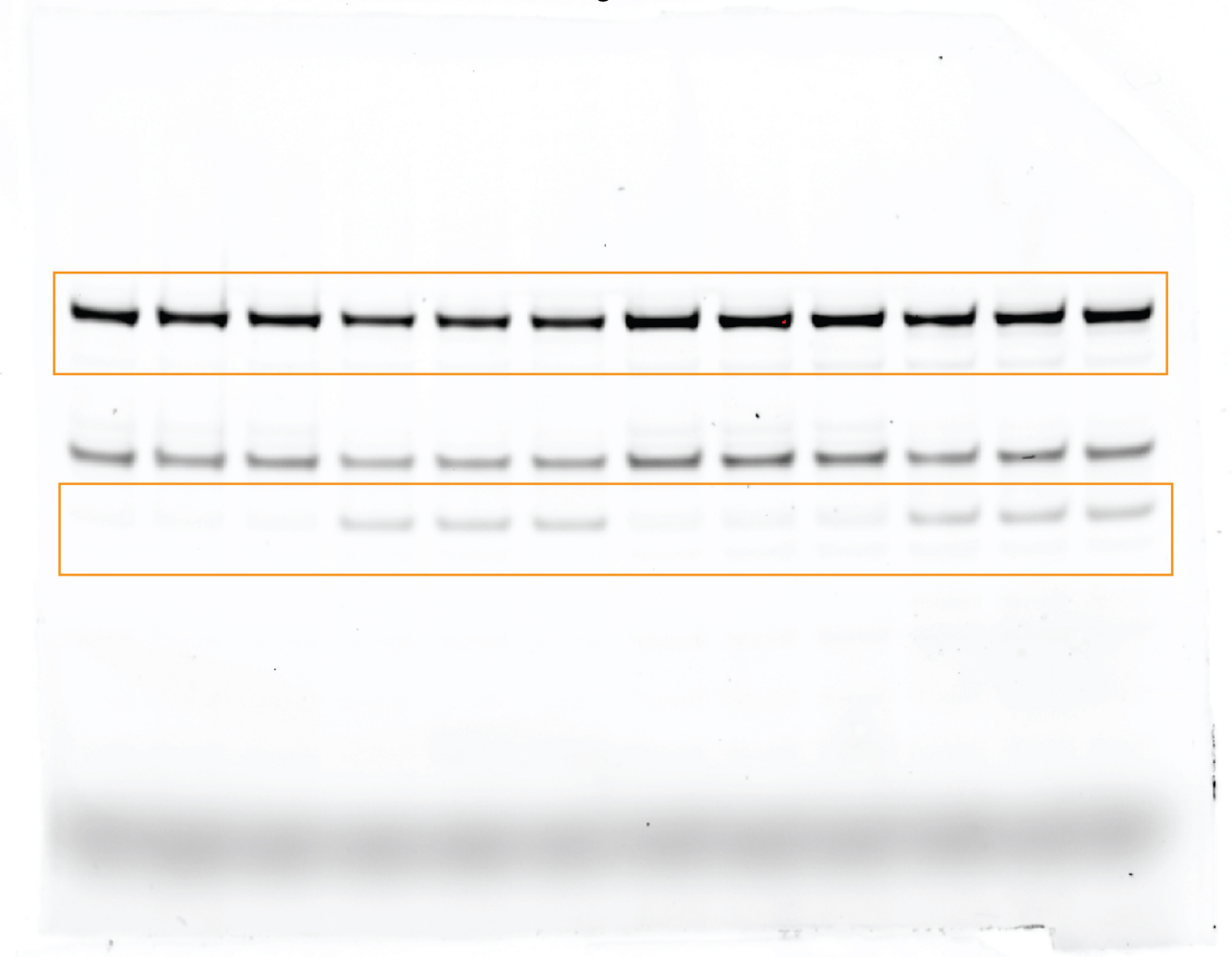

Figure 1C

Supplement: SourceData F1 — is the source file for Fig. 1. [file JCB_202309015_SourceDataF1.pdf]

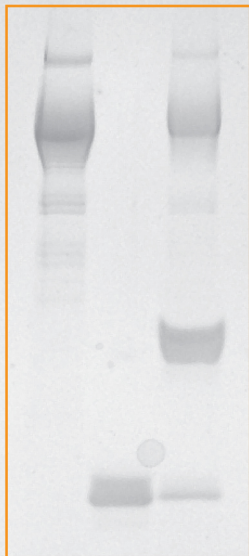

Supplement: SourceData F2 — is the source file for Fig. 2. [file JCB_202309015_SourceDataF2.pdf]

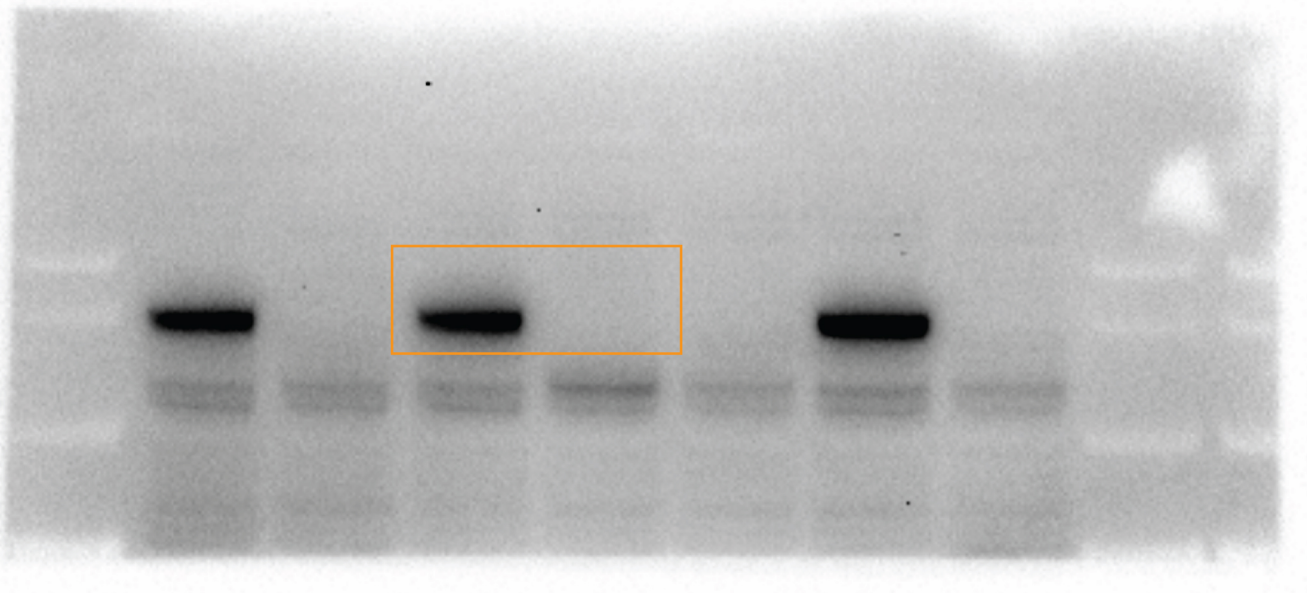

Figure S1A - Rubicon expression (see figure S4 for MW standards)

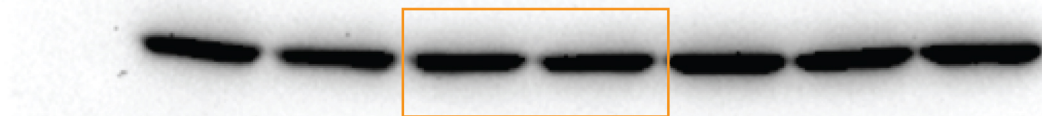

Figure S1A - Actin

Supplement: SourceData FS1 — is the source file for Fig. S1. [file JCB_202309015_SourceDataFS1.pdf]

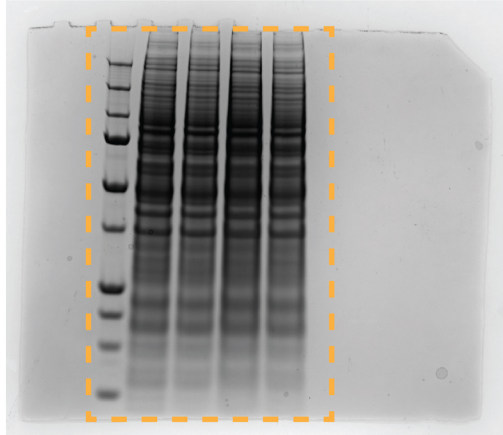

Figure S4A

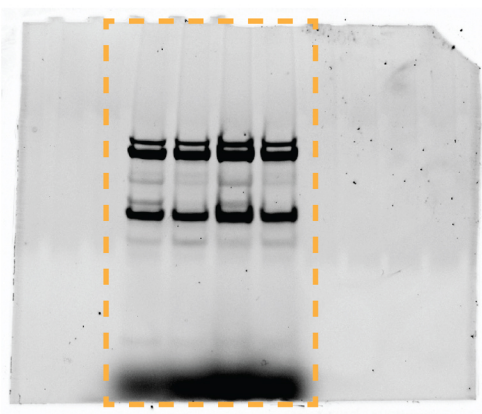

Figure S4A

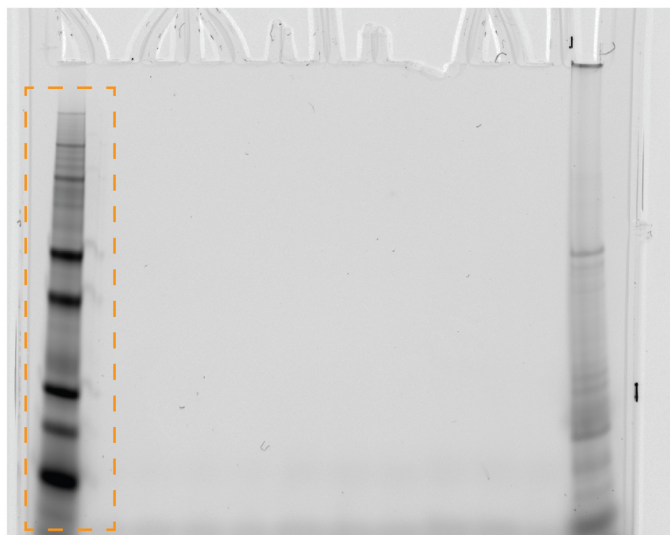

Figure S4B

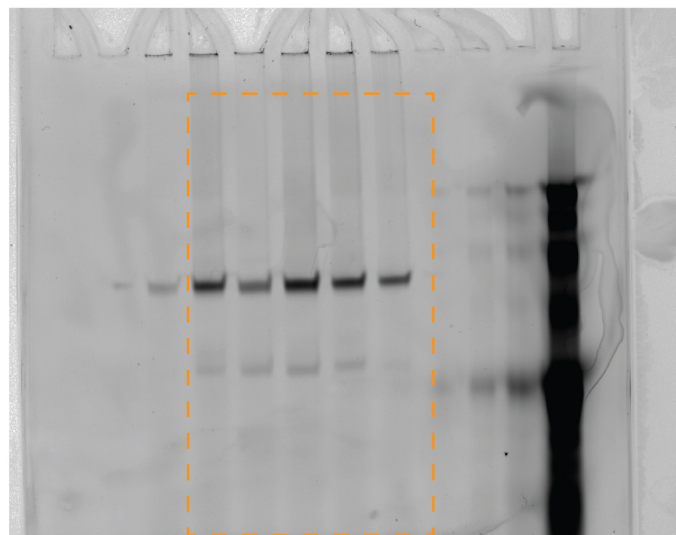

Figure S4B

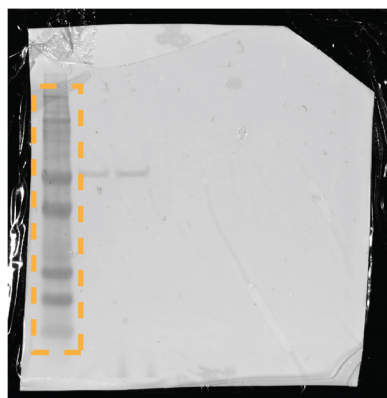

Figure S4C

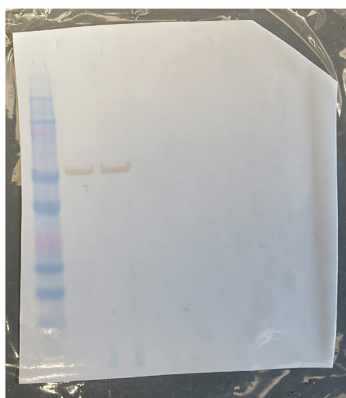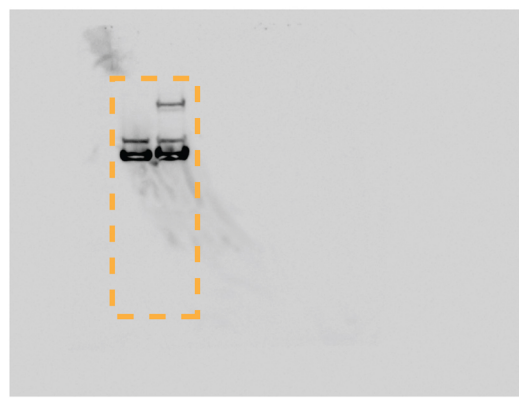

Figure S4C

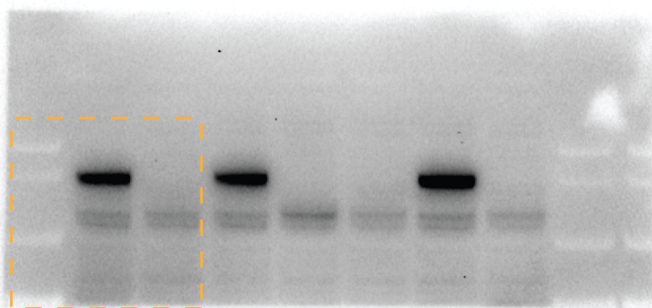

Figure S4D

Supplement: SourceData FS4 — is the source file for Fig. S4. [file JCB_202309015_SourceDataFS4.pdf]
