# Supplementary material for: A RAB7A phosphoswitch coordinates Rubicon Homology protein regulation of Parkin-dependent mitophagy
Source: J Cell Biol. 2024 May 10;223(7):e202309015. doi: 10.1083/jcb.202309015 (PMC11090050; doi:10.1083/jcb.202309015)
Supplement: SourceData F5 — is the source file for Fig. 5. [file JCB_202309015_SourceDataF5.pdf]

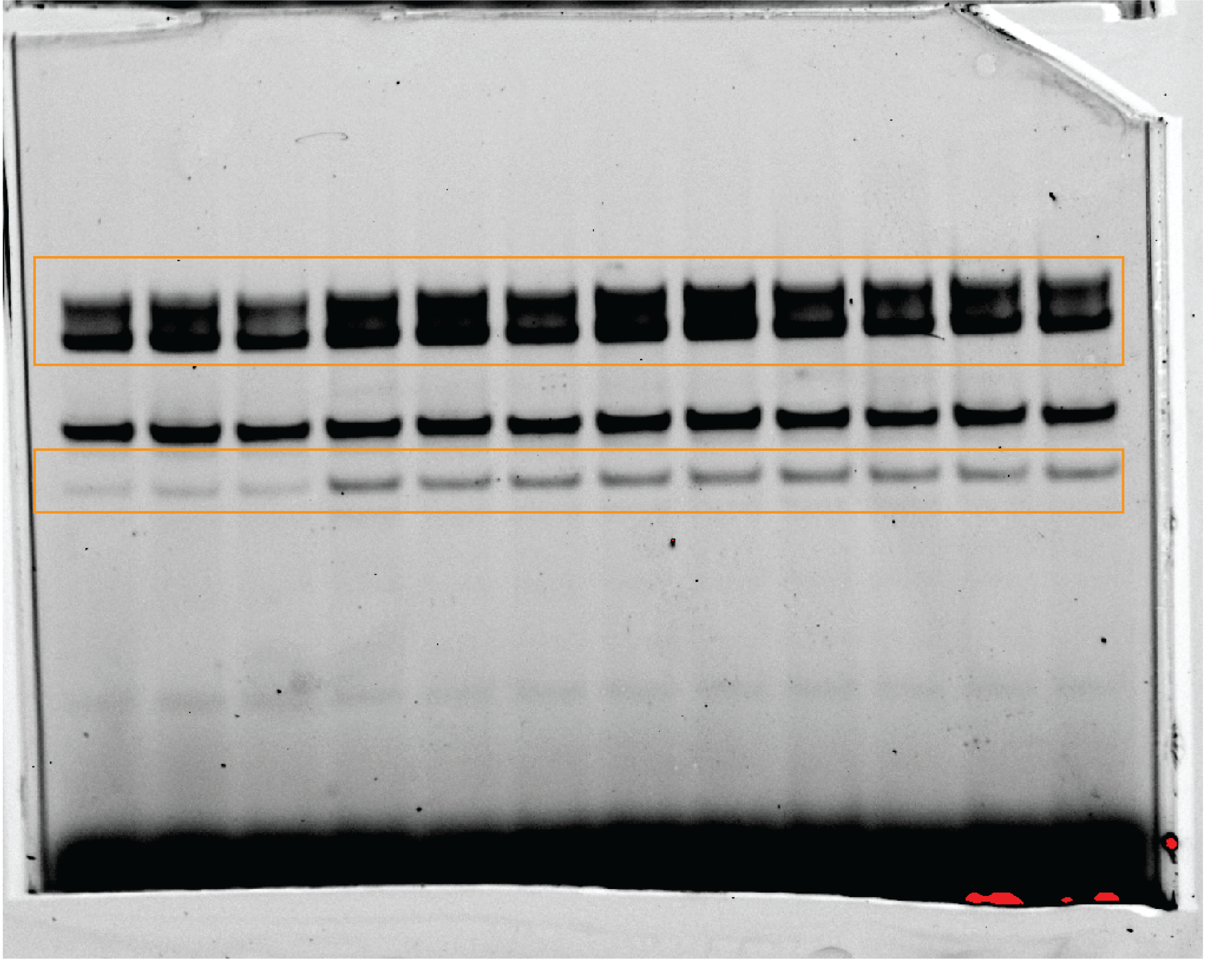

figure 5A - WT

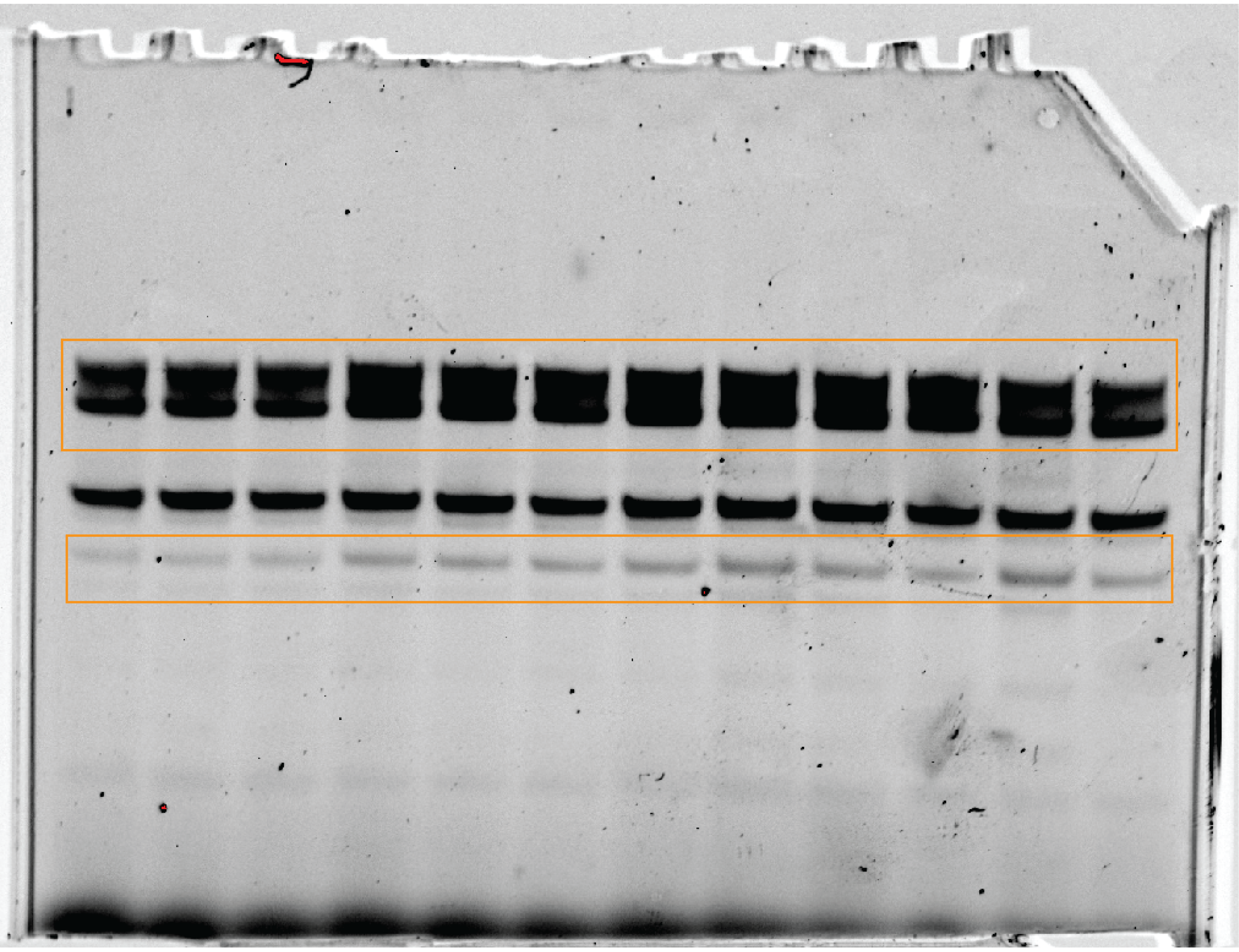

figure 5A - Pacer

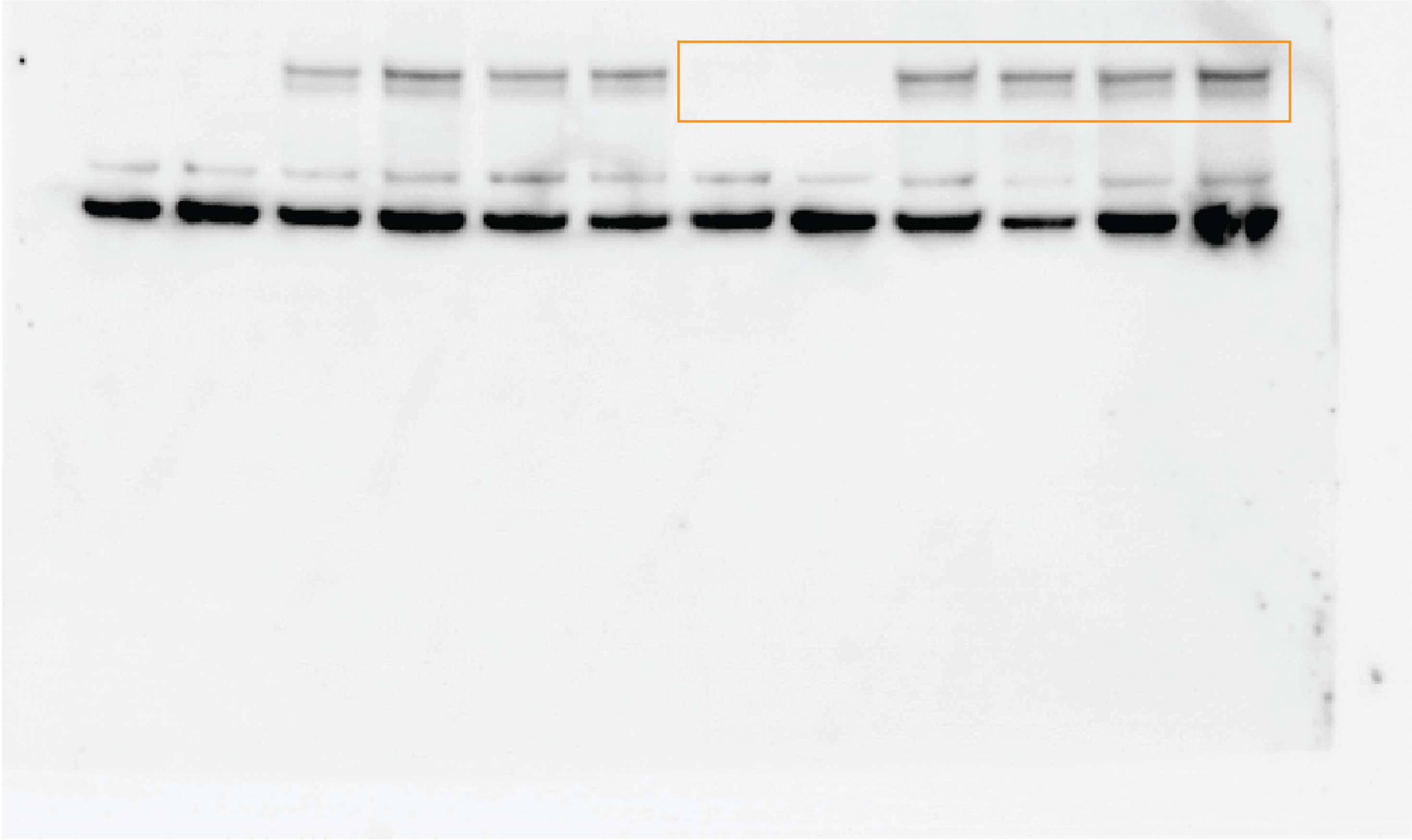

figure 5C - Pacer expression

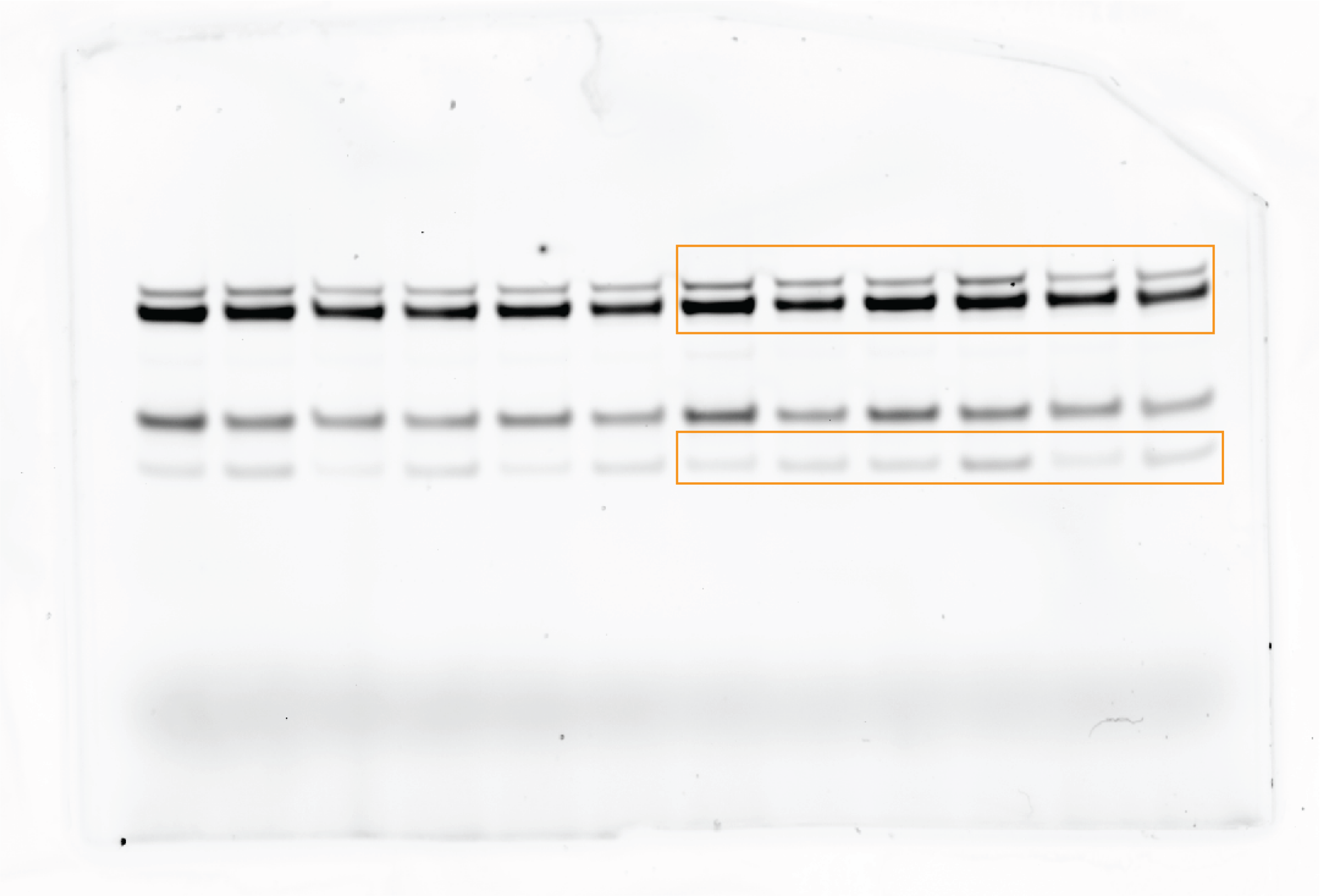

figure 5C - Su9-halo processing

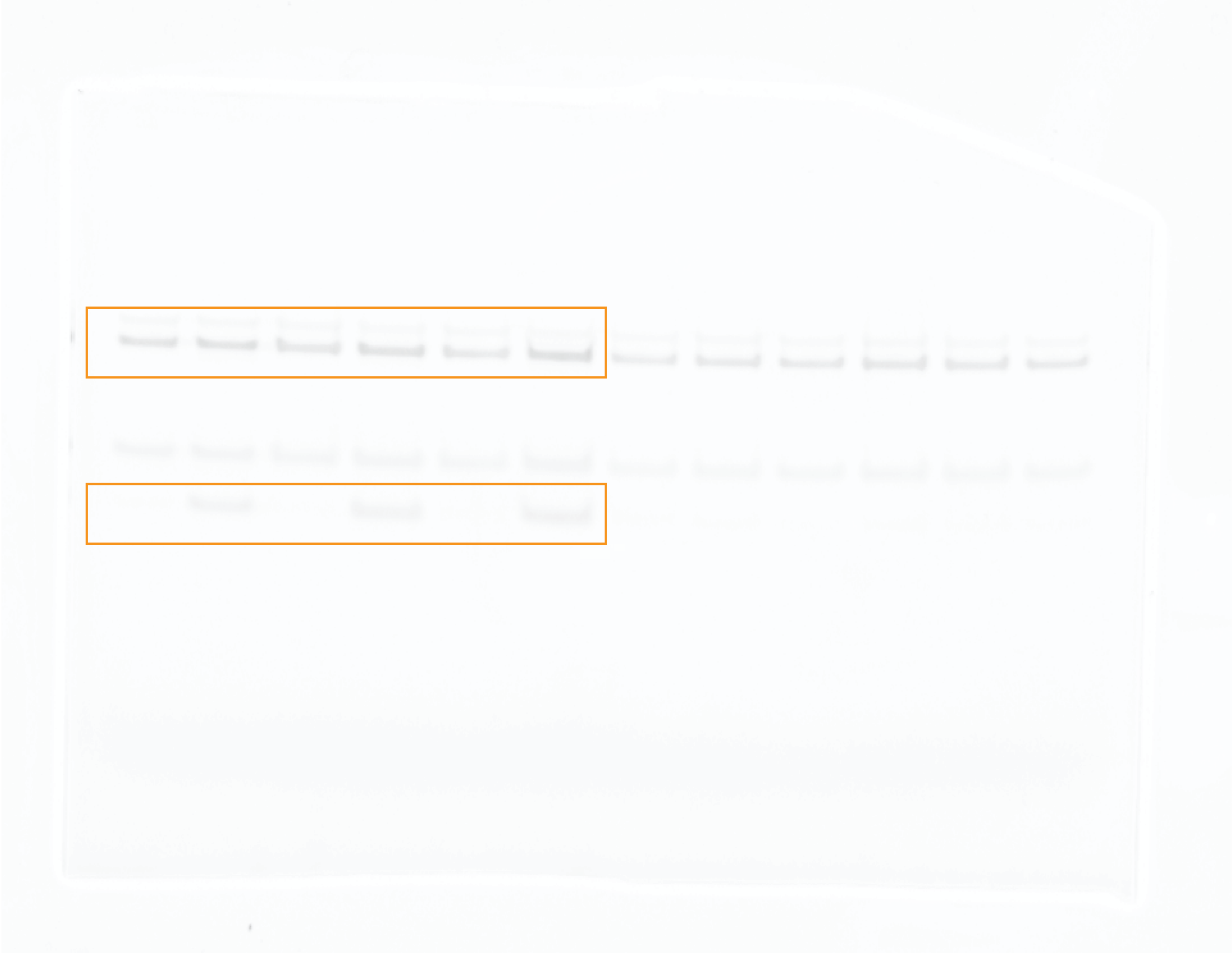

figure 5E - original exposure

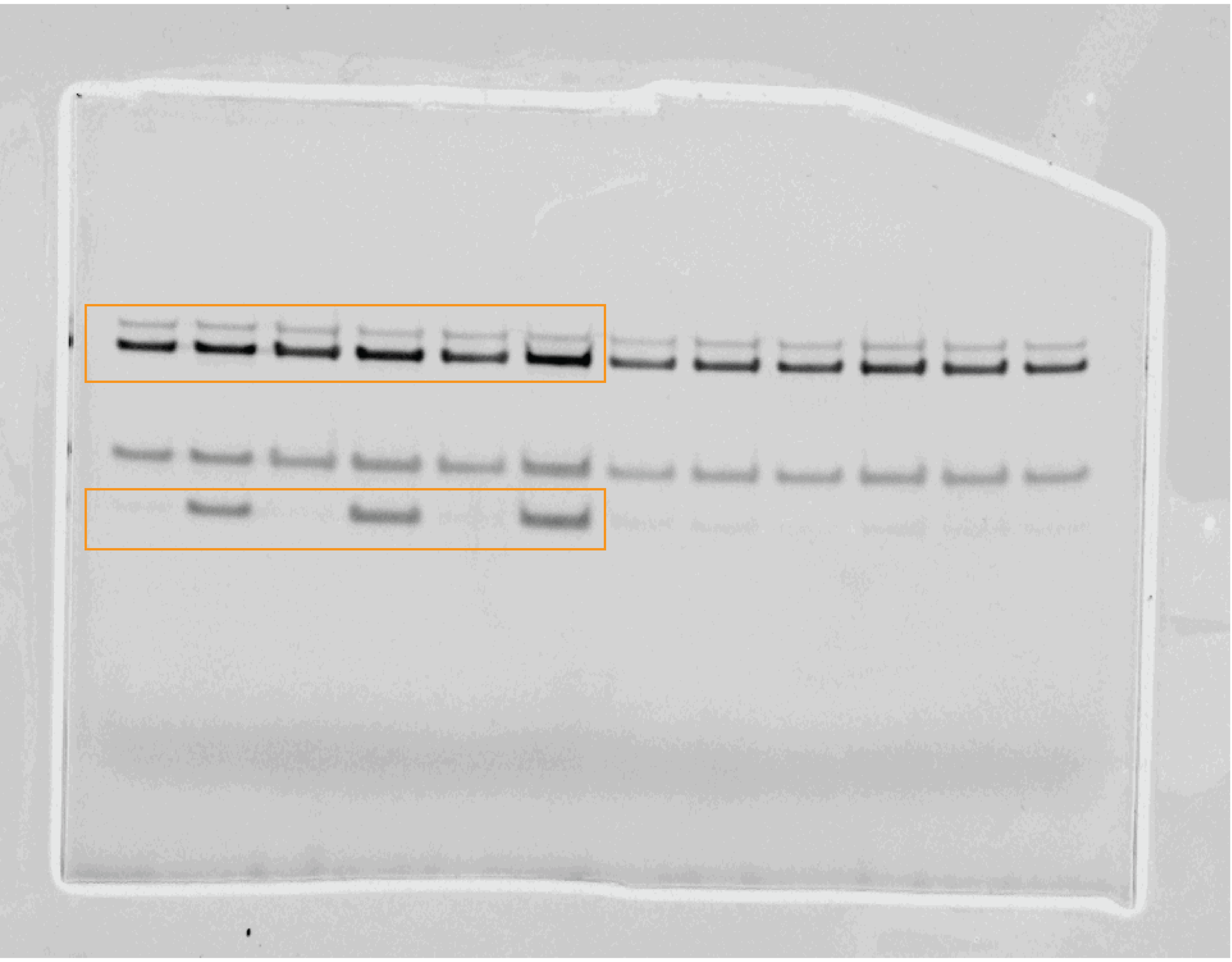

figure 5E - increased brightness

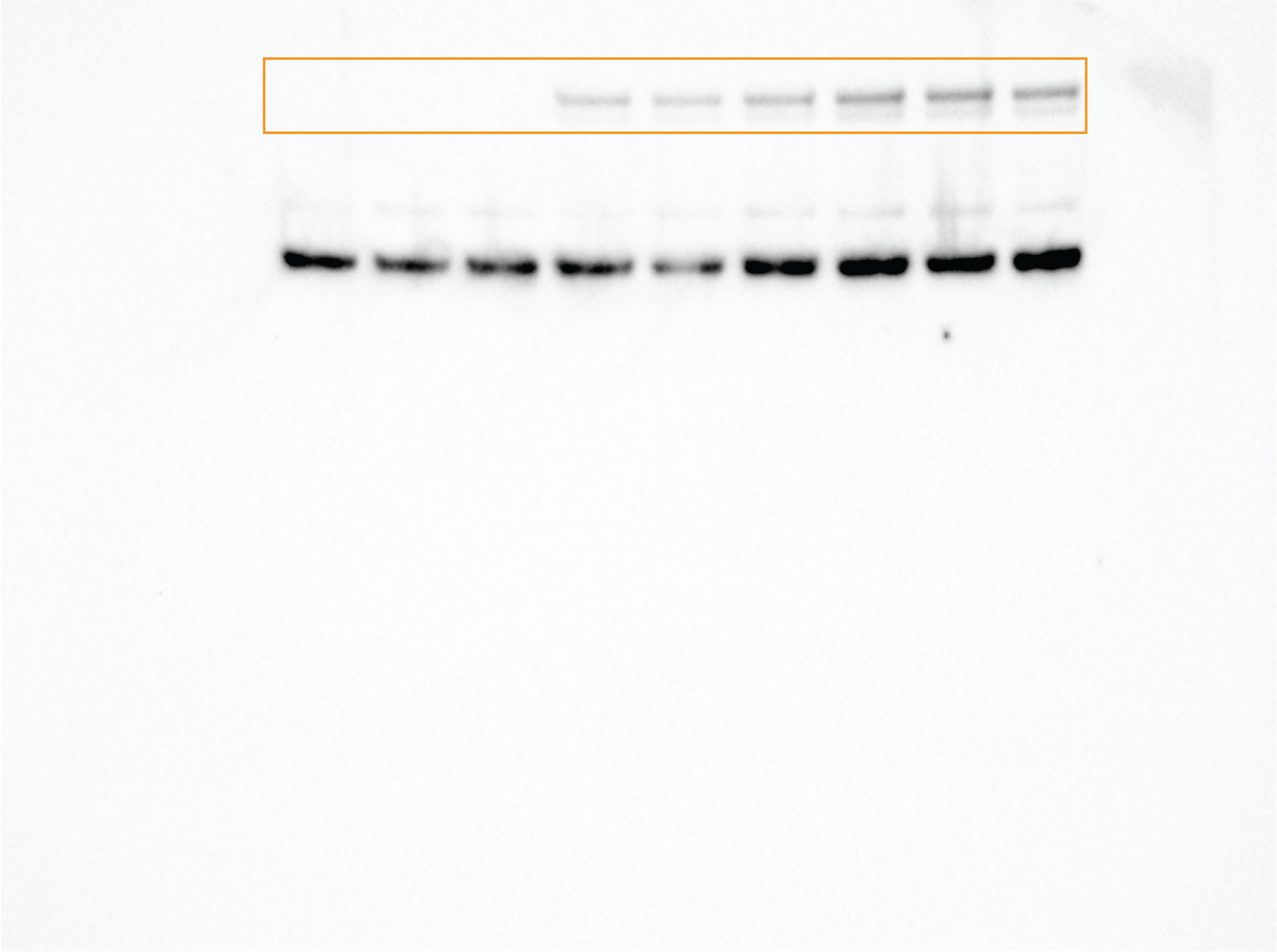

figure 5g - Pacer expression

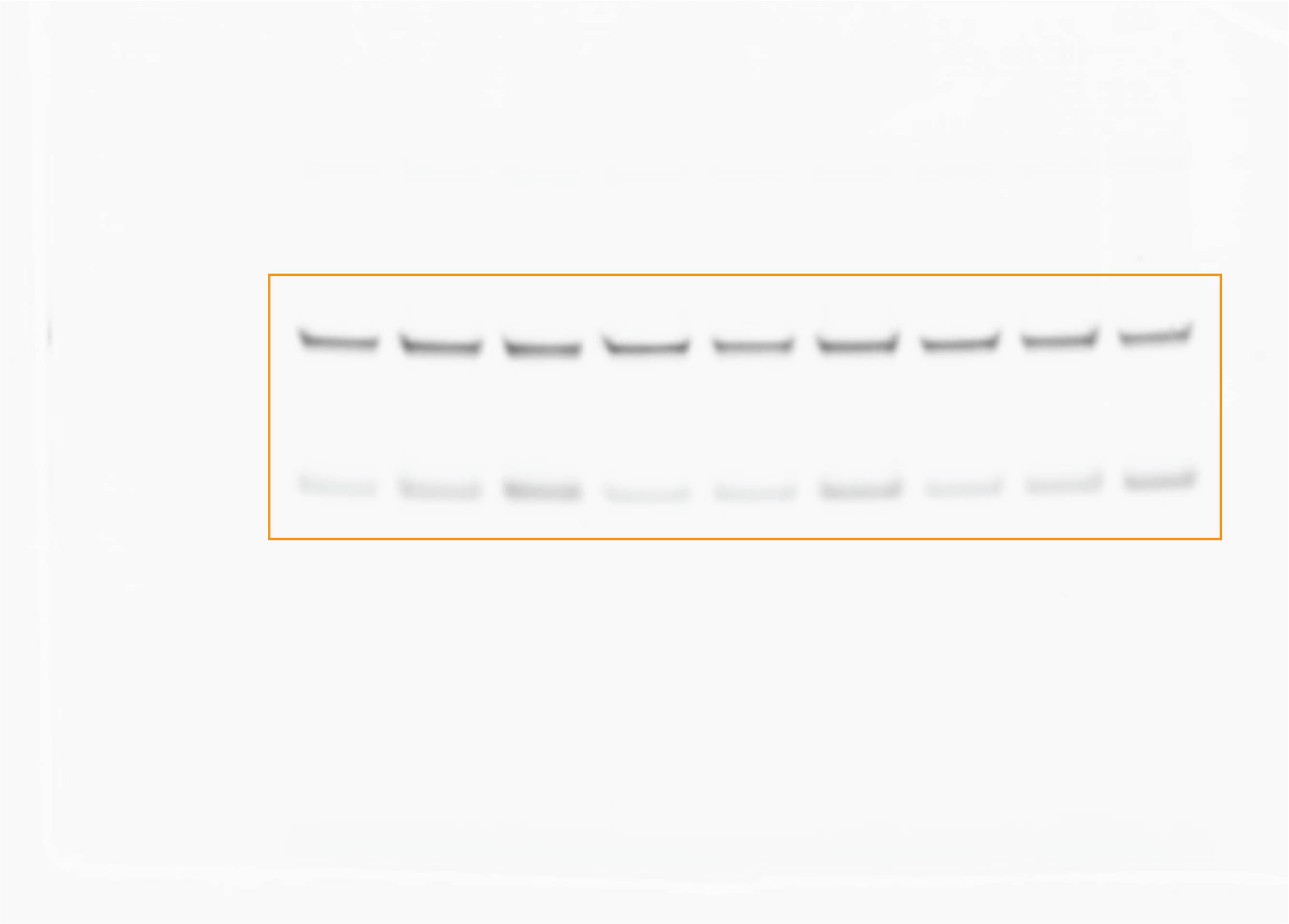

figure 5g - LC3-halo processing
